# Simvastatin protects high glucose-induced H9c2 cells from injury by inducing autophagy

**DOI:** 10.1080/13880209.2020.1839512

**Published:** 2020-11-09

**Authors:** Lusha E, Hong Jiang

**Affiliations:** a Department of Cardiology, Inner Mongolia People’s Hospital, Hohhot, China; b Department of Cardiology, Renmin Hospital of Wuhan University, Wuhan, China

**Keywords:** Myocardial protection, cardiomyocyte injury, diabetes-associated cardiovascular diseases

## Abstract

**Context:**

Simvastatin is the first line therapeutic drug for coronary heart disease and atherosclerosis. The protective effect mechanism of simvastatin on cardiomyocytes is unclear.

**Objective:**

This study explores the effect of simvastatin on high glucose induced cardiomyocyte injury and the role of autophagy during the process.

**Materials and methods:**

H9c2 cells were incubated with different doses of glucose (0, 50, 100, 200 mM) for 24 h to verify the glucose induced injury. The H9c2 cells were pre-treated with simvastatin at different dosages (0, 0.1, 0.5, 1 μM) for 30 min to rescue the injury followed by the autophagy evaluation. 3-MA was used as an autophagy inhibitor to confirm the role of autophagy in simvastatin treated process. CCK-8 assay, FACS assay, confocal microscopy, western blotting and immunofluorescence analysis were conducted to evaluate the high glucose induced injury or protective effects of simvastatin in H9c2 cell line.

**Results:**

High glucose dramatically decreased H9c2 cell viability (0 mM, 0.58 ± 0.09%; vs. 50 mM, 8.67 ± 0.43%; 100 mM, 16.1 ± 3.56%; 200 mM, 32.9 ± 2.63%), induced significant cell apoptosis (0 mM, 0.96 ± 0.16%, vs. 50 mM, 7.00 ± 0.63%; 100 mM, 12.9 ± 0.78%; 200 mM, 21.8 ± 1.17%) and suppressed cell autophagy. Simvastatin decreased apoptosis and attenuate injury by decreasing cell apoptosis ratio, elevating Bcl-2 expression while decreasing Bax and caspase-3 protein expressions. Meanwhile, simvastatin restored the autophagy depicted by western blotting with increased ATG-5, Beclin1 and LC3II/LC3I protein expression and decreased p62 expression, as well as immunofluorescence with elevated LC3 fluorescence density.

**Discussion and conclusions:**

The myocardial protective effect mediated by autophagy activated by simvastatin to some extent elucidated the mechanism of the protective effect of simvastatin on H9c2 cell injury, which provided a certain theoretical basis for the clinical application of simvastatin in the treatment of cardiovascular diseases. In addition, we speculate that simvastatin may be used for diabetes associated cardiovascular diseases.

## Introduction

Statins is a category of agents that inhibit 3-hydroxy-s-methylglutaryl-coenzyme A (HMG-CoA) reductase. Statins block cholesterol biosynthesis, therefore lowing blood cholesterol levels, modulating arterial myocytes biological characteristics and functions, and therefore are widely applied to lower plasma cholesterol and treat atherosclerotic disease (Wei et al. 2013). In recent years, several studies suggested that statins have a modulating effect for the regulation of autophagy and apoptosis (Yang et al. 2010; Ghavami et al. 2011). Simvastatin is the first line therapeutic drug for coronary heart disease and atherosclerosis. Researches indicated that simvastatin can also increase autophagy in coronary arterial myocytes by inhibition of major negative autophagy regulator Racl-mammalian target of rapamycin (mTOR), a pivotal AMPK/Akt downstream target (Wei et al. 2013). However, the effect and mechanism of autophagy in simvastatin treated high glucose induced injury remains unclear.

Hyperglycaemia is the manifestation of metabolism imbalance. It is noted that hyperglycaemia is a vital detrimental factor of cardiovascular events, and multiple patients with diabetes are experiencing cardiomyocyte hypertrophy and injured heart function (Kuwabara et al. 2014). Autophagy is a dynamic catabolic process to clear long-lived, damaged and aggregated cellular components by delivering cytoplasmic material to the lysosomal machinery for degradation and recycling, which maintains and balances the cellular and organismal homeostasis (Tan et al. 2018). Autophagic activity may decrease along with the increase of age and may explain many features of age-related cardiac dysfunction. Altered autophagy may lead to metabolic disorders. Autophagy deficiency is implicated in metabolic disorders, such as obesity, insulin resistance, diabetes mellitus and atherosclerosis (Jing et al. 2018). Moreover, autophagy destruction may also contribute to low grade chronic inflammation, adipose tissue deterioration and lipid accumulation in obesity (Soussi et al. 2016). Autophagy is characterized with several protein markers. ATG5 is an early autophagosomal marker which transiently localized to punctate on mitochondria, followed by the late autophagosomal marker, light chain-3 (LC3) (Bento et al. 2016). Inducible deletion of ATG5 in mice represents the loss of autophagy, which leads to heart failure (Nakai et al. 2007). The amount of total cellular LC3 and its substrate p62 inversely correlated with autophagic flux, which can be quantified by immunoblotting assay or flow cytometry (Mizushima and Yoshimori 2007). Previous study indicated that the Beclin-1, ATG 5-12, p62 in patients were notably depleted under ischaemic stress, and the variation of p62 levels correlated significantly associated with changes in Beclin-1, ATG 5-12, LC3-I and LC3-II, which indicated the autophagy proteins deficiency and the increase of autophagic flux (Jahania et al. 2013).

Autophagy inhibition by 3-methyladenine (3-MA) is one of the autophagy interventions, therefore 3-MA was used as an inhibitor in our assay (Jahania et al. 2013). In this study, we explored the role of autophagy in simvastatin treated H9c2 cardiomyoblasts under high glucose induced injury. To our knowledge, whether simvastatin alleviates cardiac injury through increase the myocardial autophagy in cardiomyocytes has not been elucidated. Here, we utilized H9c2 cell and flow cytometry, western blot and other methods to figure out the potential effect of simvastatin to attenuate cardiac injury under hyperglycaemia by inducing protective autophagy.

## Materials and methods

### Cell culture

H9c2 cells were purchased from Cell Bank of the Chinese Academy of Sciences (Shanghai, China) and then cultured in DMEM-low glucose (HyClone) supplemented with 10% foetal calf serum at 37 °C in an incubator with 5% CO_2_. The medium was replaced every day. When confluence reached 80–90%, the cells were seeded in six-well cell culture plate with 5 × 10^5^ cells each. Twenty-four h later, different concentrations of d-glucose (Sigma) was added in cell with final concentration at 0, 50, 100, 200, 300 and 400 mM for 24 h, respectively. For simvastatin (SVT) (Sigma) treatment, H9c2 cells were pre-treated with different concentrations of simvastatin for 30 min. The concentrations of simvastatin were 0, 0.1, 0.5 and 1 μM, respectively, and then cultured with different concentration of glucose (0, 200 mM) for 24 h. To study the involvement of autophagy, 1 mM 3-MA was added together with _D_-glucose after 30 min of simvastatin pre-treatment. All experiments were repeated for at least three times.

### CCK-8 assay for cell viability

Cell viability in H9c2 cells was determined by cell counting kit- 8 solution (CCK-8) assay (Dojindo Laboratories, Shanghai, China). Briefly, H9c2 cells were cultured in 96-well plates at 1 × 10^5^ cells/mL for CCK-8 assay. After treatment with glucose or drugs, the cells were incubated with CCK-8 solution (10 µL) for an hour and the steps were strictly followed the manufacture’s instruction.

### Flow cytometric analysis for apoptosis

Fluorescence-activated cell sorting (FACS) with Annexin V-FITC/PI (4 A Biotech, Beijing, China) staining was utilized to quantitatively examine the apoptosis of cells and performed according to the manufacturer’s instructions. Briefly, H9c2 cells were washed with phosphate buffer solution (PBS) twice and resuspended in binding buffer. Cells were then incubated with 5 µL Annexin V- FITC/PI for RT in dark for 15 min. Flow cytometer (ACEA) was then used to analyze the cells from each sample.

### Immunofluorescence for protein expression

For cell immunofluorescence, cells treated with different test reagents were fixed by 4% paraformaldehyde on chamber slides for 30 min at room temperature. Fixed cell was then permeated in 0.5% Triton X-100/PBS for 20 min and blocked by 2% goat serum in PBS for an hour. The primary antibodies of LC3 (1:100) (Sanying, Wuhan, China) and specific second FITC-labelled IgG antibodies were incubated with cells continuously. Finally, the images were examined with confocal microscope.

### Western blotting for protein expression

The H9c2 cells were lyzed in RIPA buffer with protease inhibitors and phosphatase inhibitors for 5 min on ice. BCA analysis kit (Biocolors, Shanghai, China) was utilized to detect the protein concentrations. Equal amount of protein extraction (20 μg) was loaded into an electrophoresis apparatus of 8-12% sodium dodecyl sulphate-polyacrylamide gels (SDS-PAGE) and, after the completion of electrophoresis, the blot was transferred onto a polyvinylidene fluoride (PVDF) membrane (Millipore) followed by timed incubation with the selected primary antibodies anti-Beclin1 (Proteintech, 11306-1-AP), anti-LC3 (Proteintech, 18725-1-AP), anti-p62 (Proteintech, 18420-1-AP), anti-Bcl-2 (Proteintech, 26593-1-AP), anti-Bax (Proteintech, 50599-2-Ig), anti-cleaved-caspase-3 (Proteintech, 19677-1-AP), anti-GAPDH (Proteintech, 10494-1-AP) under shaking overnight at 4 °C, and subsequently incubated with secondary antibodies goat anti-rabbit/mouse IgG-horseradish peroxidase (HRP) (Sanying, Wuhan, China) for 2 h at room temperature. Finally, the bands were detected using an enhanced chemiluminescence (ECL) detection system.

### Statistical analysis

All data were expressed as means ± standard deviation. Statistical analysis was performed using One-way analysis of variance (ANOVA) between each group and Tukey’s test for *post hoc* test was followed. *p* value of < 0.05 was deemed to be significant.

## Results

### High glucose stimulation leads to apoptosis in H9c2 cells

**Figure 1.**
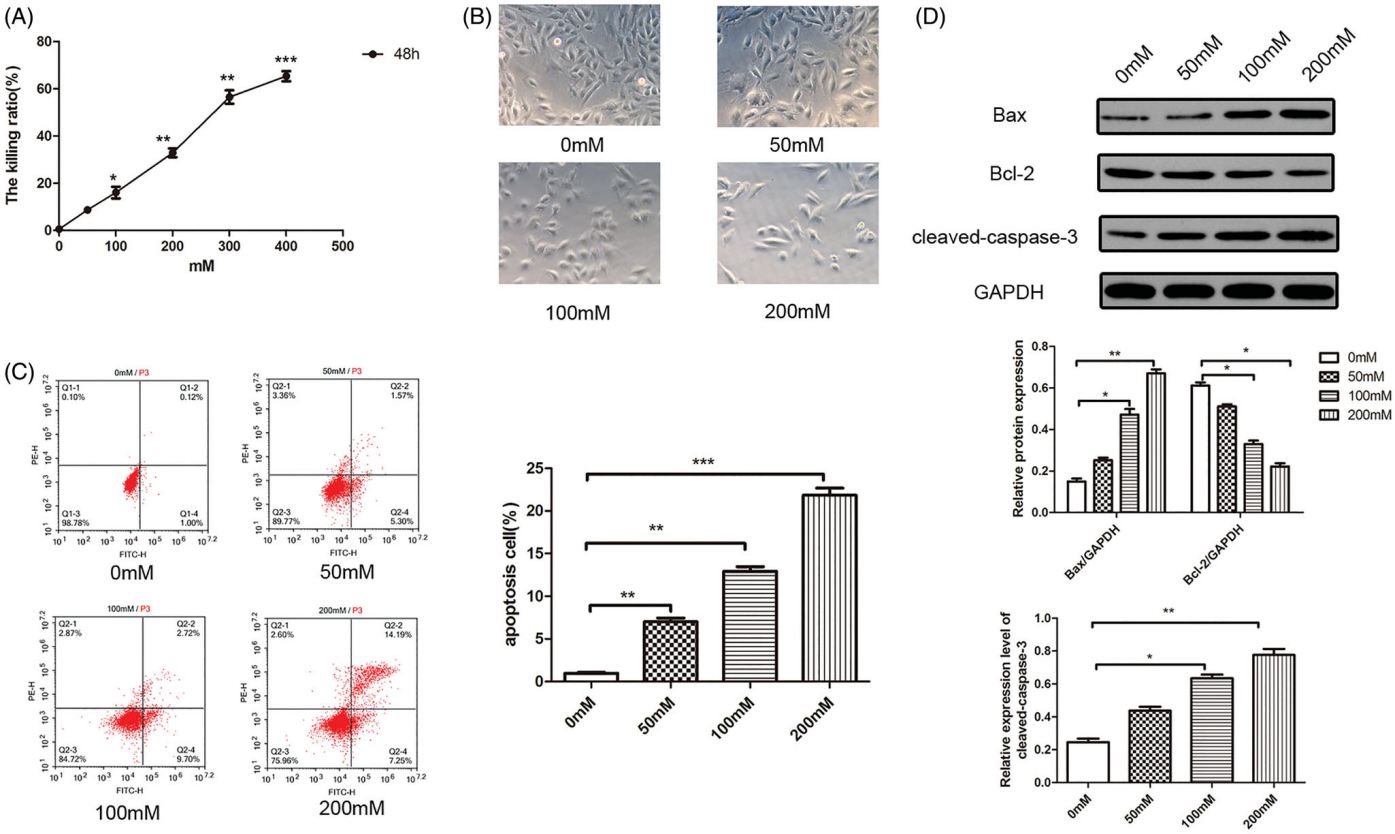
High glucose stimulation leads to apoptosis in H9c2 cells. (A) CCK8 assay was used to detect the proliferation of H9c2 cells stimulated by different concentrations of high glucose. (B) Microscopic photo of cell morphology under stimulation of different concentrations of high glucose. (C) Flow cytometry for apoptosis under stimulation of different concentrations of high glucose. (D) Western blotting detection for apoptosis-related molecules under stimulation of different concentrations of high glucose. ***p < 0.001 as compared with the 0 mM group. The experiments were run three times.

To examine the detrimental effect of high-glucose on H9c2 cells, a cohort of H9c2 cells were treated with different dosages of glucose. As shown in Figure 1(A), high glucose incubation decreased the cell viability in a dose dependent manner, and among 0-200 mM glucose treated groups, the killing ratio increased in a near liner manner (0 mM, 0.58 ± 0.09%; 50 mM, 8.67 ± 0.43%; 100 mM, 16.1 ± 3.56%; 200 mM, 32.9 ± 2.63%). 300–400 mM glucose treated groups resulted in too much apoptosis, which is not suitable for the observation of autophagy increased by simvastatin. Therefore, four groups of 0–200 mM dosages were subjected to following tests.

Moreover, the cells observed under microscope were decreased obviously with the increment of glucose dosages (Figure 1(B)). High glucose incubation also accelerated cell apoptosis as evidenced by FACS assay (Figure 1(C)) and apoptosis cell ratios were increased along with the elevation of glucose concentrations (*p* < 0.05). Consistent with the results of FACS assay, the apoptosis associated protein expressions were altered under the treatment with high glucose, in which Bax and cleaved-caspase-3 protein expression were increased while Bcl-2 protein expression was decreased in a dose dependent manner (*p* < 0.05).

### The autophagy was inhibited after high glucose stimulation

**Figure 2.**
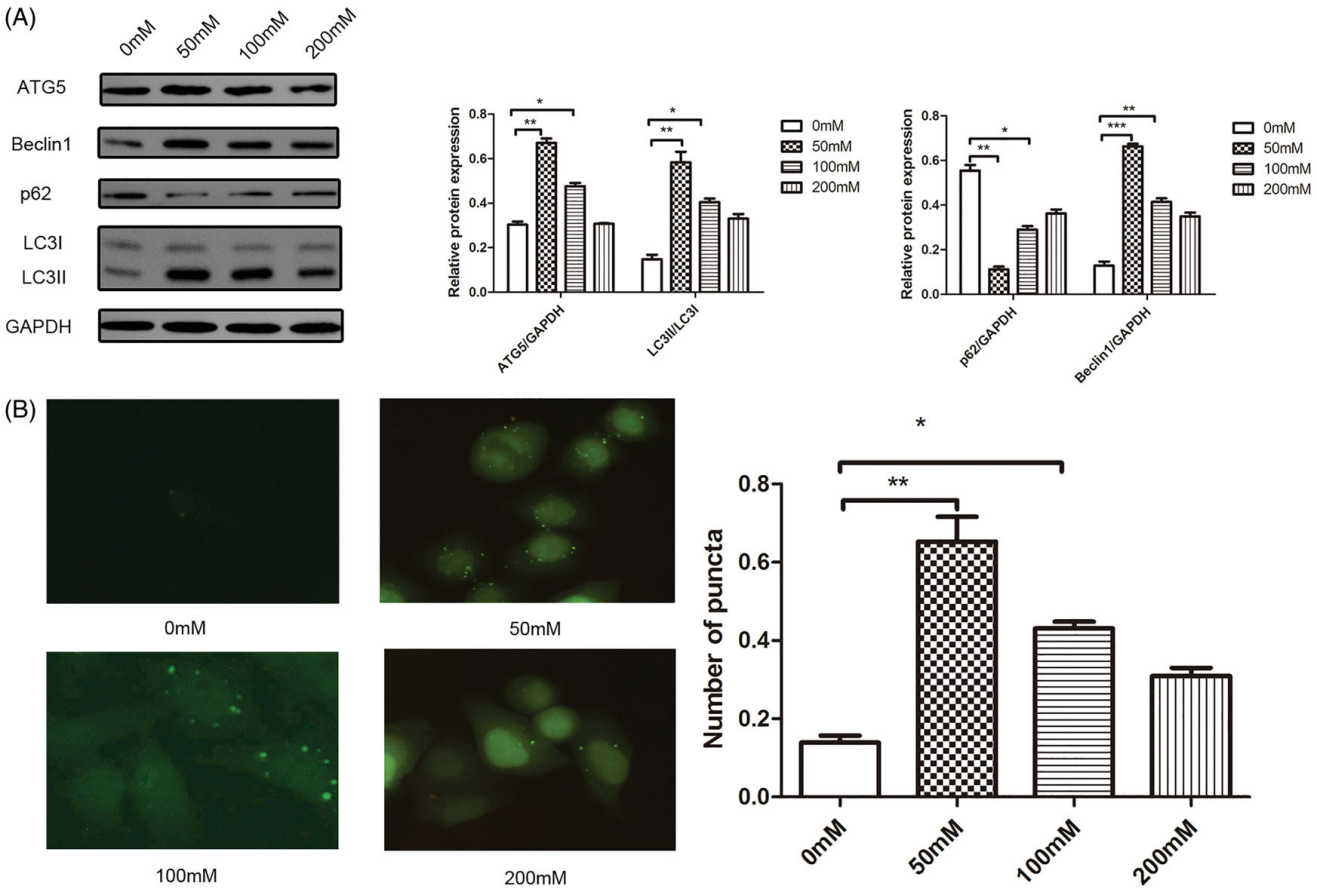
The autophagy was inhibited after high glucose stimulation. (A) Western blotting detection for autophagy related molecules after high glucose stimulation. (B) Immunofluorescence detection for LC3 expression after high glucose stimulation. *p < 0.05, **p < 0.01 as compared with the 0 mM group. The experiments were run three times.

To test if high glucose treatment contributes to the autophagy decrease, characteristic protein expressions of autophagy were quantified by western blotting assay. According to the results (Figure 2(A)), the autophagy was inhibited with glucose treatment, the expressions of ATG5, Beclin and LC3II/LC3I were at max when glucose concentration was at 50 mM while p62 was at lowest. With the increment of glucose concentration, the protein expressions were adversely altered. Consistently, under microscope observation (Figure 2(B)), the mean fluorescence of LC3 was also altered as western blotting assay result showed, which indicated that the total LC3 protein expression was decreased under glucose toxicity and autophagy ability also decreased. Among the tested groups, the 200 mM glucose induced injured group exhibited the highest apoptosis ratio and lowest autophagy, therefore this concentration was applied to the following test.

### Simvastatin protects high glucose-induced H9c2 cells from injury

**Figure 3.**
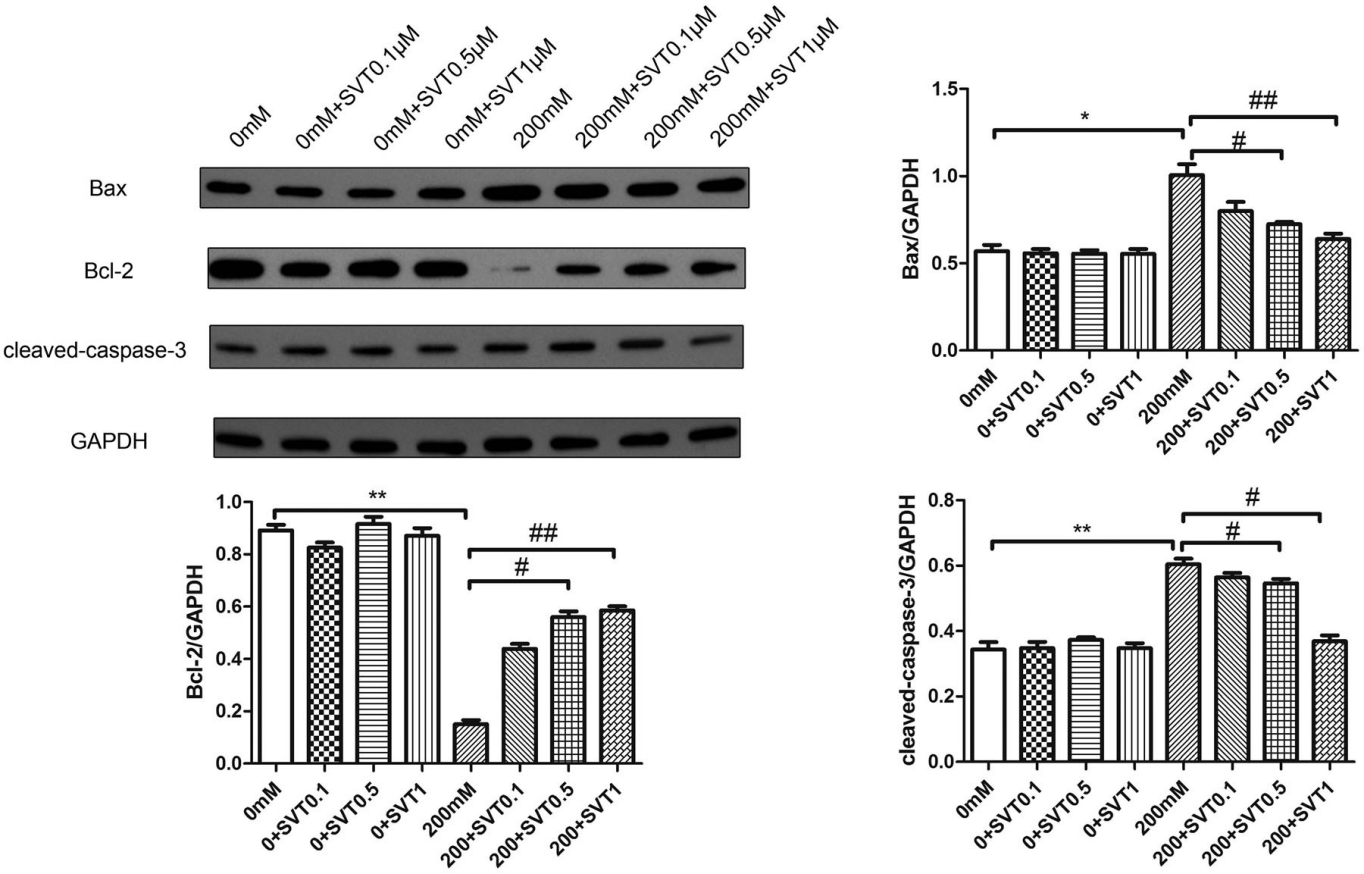
Simvastatin protects high glucose-induced H9c2 cells from injury. Western blotting detection of apoptosis related molecules with the treatment of simvastatin. *p < 0.05, **p < 0.01 as compared with the 0 mM group. #*p* < 0.05, ##*p* < 0.01, as compared with the 200 mM group. The experiments were run three times.

Bax, cleaved-caspase-3 and Bcl-2 are apoptosis related factors. To explore the therapeutic effect of simvastatin in high glucose-induced injury, different dosages of simvastatin were studied in high glucose treated H9c2 cells. According to the results (Figure 3), despite of the increment of dosages, simvastatin did not influence the apoptosis related factor’s protein expression in high glucose absent groups. However, after high glucose stimulation (200 mM), Bax and caspase-3 protein expressions were up-regulated while Bcl-2 was down-regulated. With the pre-treatment of simvastatin, Bax and cleaved-caspase-3 protein expressions were down regulated in a dose dependent manner, and Bcl-2 protein expression was gradually increased (*p* < 0.001).

### Simvastatin protects high glucose-induced H9c2 cells from injury by inducing autophagy

**Figure 4.**
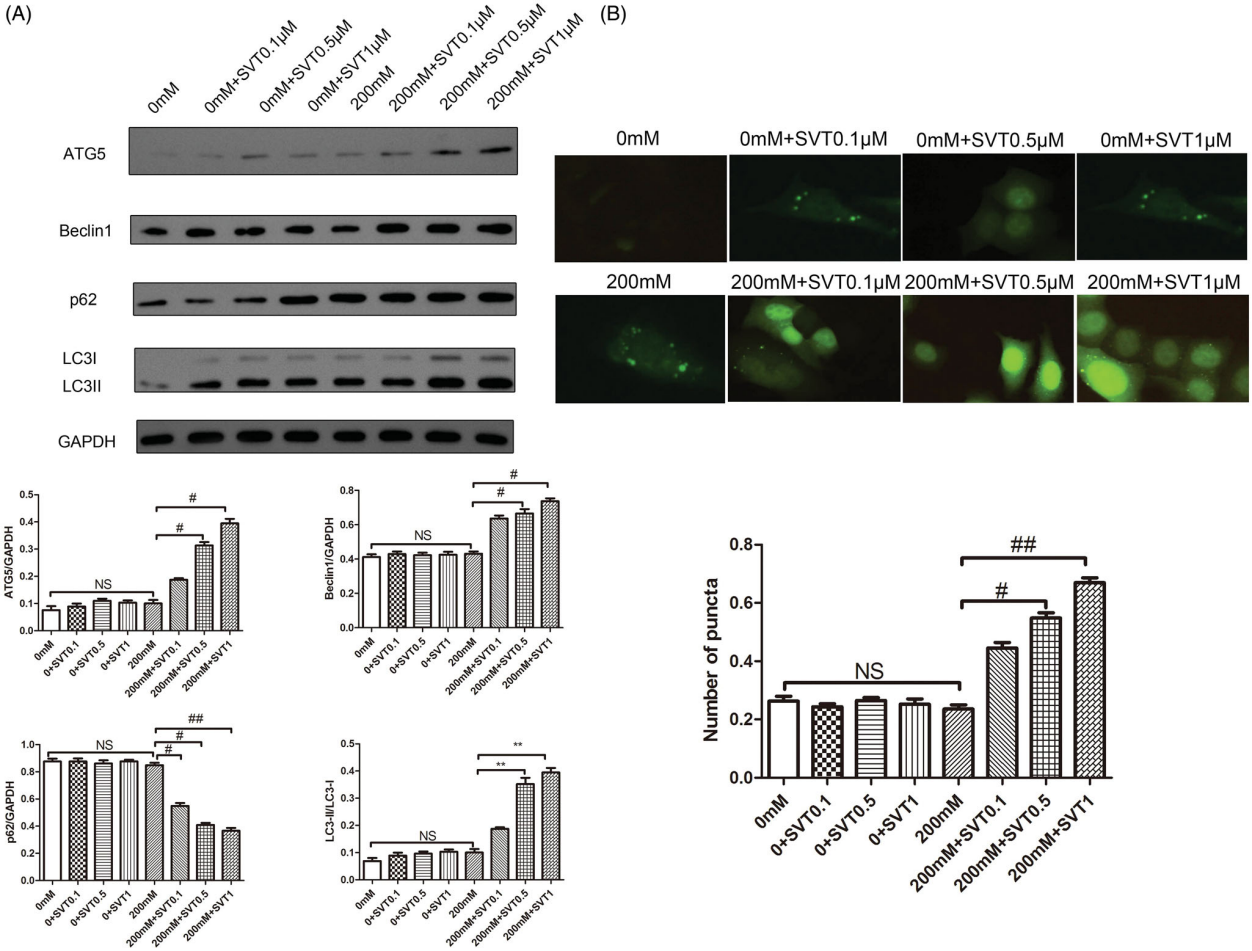
Simvastatin protects high glucose-induced H9C2 cells from injury by inducing autophagy. (A) Western blotting detection of autophagy related molecules with the treatment of simvastatin. (B) immunofluorescence detection for LC3 expression with the treatment of simvastatin. *p < 0.05 as compared with the 0 mM group. #*p* < 0.05 ##*p* < 0.01 as compared with the 200 mM group. The experiments were run three times.

Western blotting analysis was utilized to explore the potential involvement of autophagy behind simvastatin-offered protection effect against high glucose-induced injury in H9c2 cells. Results shown in Figure 4(A) suggested that cell autophagy was rested at normal state and remained compromised under the glucose stimulation as demonstrated by low amount of ATG5, Beclin1 and LC3II/LC3I protein expressions and high amount of p62 protein expressions. There was little effect on autophagy protein markers by simvastatin exhibited on normal cells. Results also indicted that autophagy was gradually up-regulated with the treatment of simvastatin under high glucose induction and performed in a dose dependent manner, in which ATG5, Beclin1 and LC3II/LC3I expressions were up-regulated while p62 was down-regulated (*p* < 0.05). Immunofluorescence test was also utilized to detect the autophagosomal maker LC3 expression (Figure 4(B)). The result obtained was in accordance with that of western blotting results.

### Autophagy inhibitor attenuates the protective effect of simvastatin on high glucose-induced H9c2 cells

**Figure 5.**
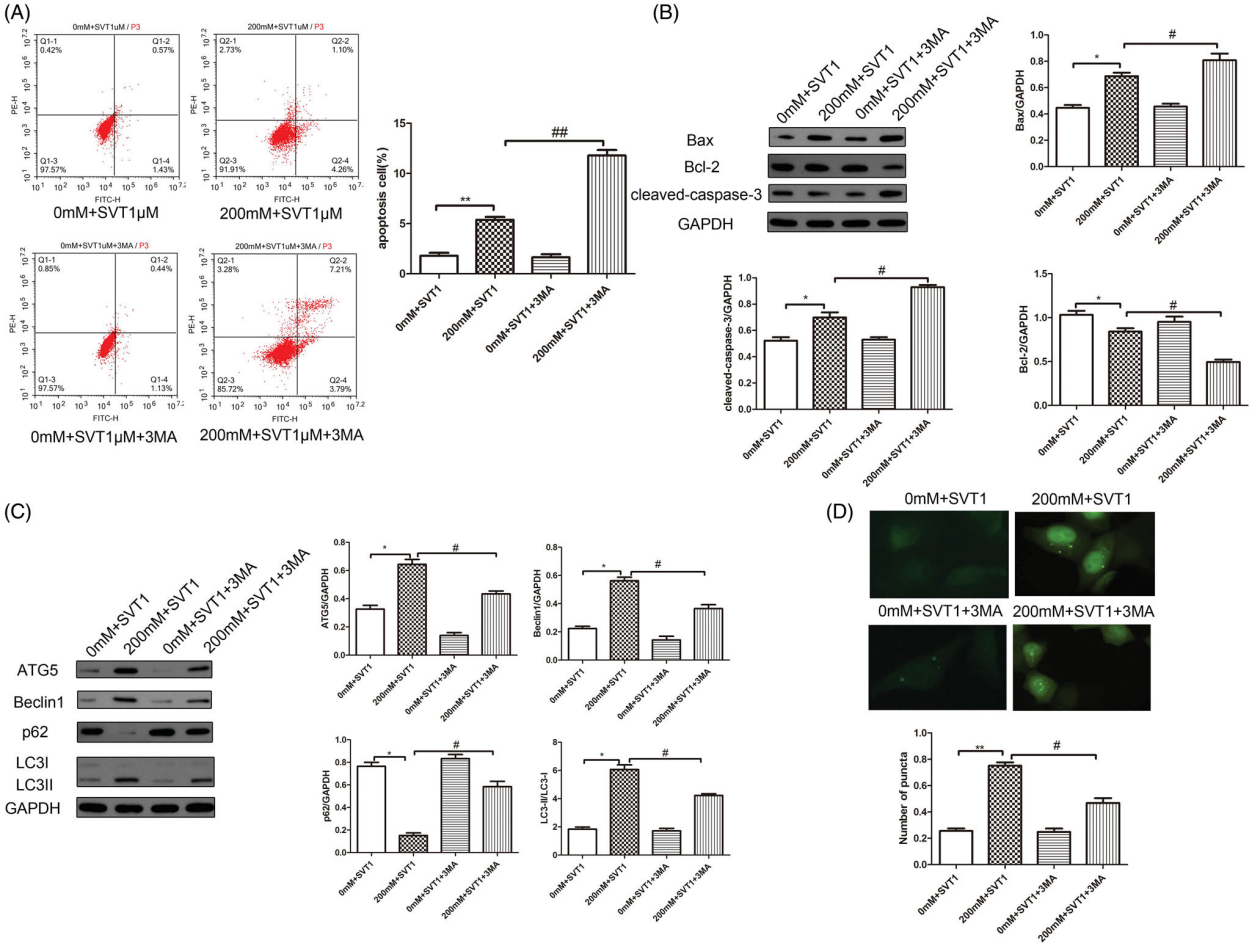
Autophagy inhibitors attenuate the protective effect of simvastatin on high glucose-induced H9c2 cells. (A) Flow cytometry for apoptosis under the treatment of simvastatin and inhibition of 3-MA. (B) Western blotting detection of apoptosis related molecules under the treatment of simvastatin and inhibition of 3-MA. (C) Western blotting detection of autophagy related molecules under the treatment of simvastatin and inhibition of 3-MA. (D) Immunofluorescence detection for LC3 expression under the treatment of simvastatin and inhibition of 3-MA. *p < 0.05, **p < 0.05 as compared with the 0 mM + SVT 1 μM group. #*p* < 0.05, ##*p* < 0.01 as compared with the 200 mM + SVT 1 μM group. The experiments were run three times.

To confirm the potential mechanism of autophagy act behind simvastatin-offered protection effect against high glucose toxicity, the H9c2 cells were incubated with or without high glucose in the presence of simvastatin or autophagosome formation inhibitor 3-MA. The FACS result (Figure 5(A)) indicated that the addition of 3-MA combined with simvastatin did not affect the normal apoptosis ratio of H9c2 cells. However, 3-MA could significantly elevate the apoptosis ratio in simvastatin treated group (*p* < 0.01). Accordingly, 3-MA also reversed the apoptosis related factor’s protein expressions which decreased by simvastatin treatment (Figure 5(B)). Western blotting assay indicated that simvastatin elevated Bcl-2 expressions but decreased by 3-MA. Simvastatin also decreased Bax and cleaved-caspase-3 expression but increased by 3-MA treatment. As for autophagy, our data indicated that 3-MA significantly inhibited autophagy evidenced by the expression of autophagic protein markers that ATG5, Beclin1 and LC3II/LC3I expression increased by simvastatin were decreased by 3-MA, along with that p62 expression decreased by simvastatin were increased by 3-MA (Figure 5(C)). Immunofluorescence staining of LC3 also revealed the same result in western blotting (Figure 5(D)).

## Discussion

Heart disease or stroke contributes to two-thirds of diabetic death. Therefore, it is important to study and cure diabetic cardiovascular complications. Stains are prescribed in cardiovascular diseases for cholesterol lowering. American Diabetes Association suggested that patients with diabetes and a history of cardiovascular disease (CVD), as well as those > 40 years of age without CVD but with CVD risk factors, should be treated with a statin regardless of their baseline LDL cholesterol concentration (Subedi et al. 2013). Simvastatin is the first line therapeutic drugs for coronary heart disease and atherosclerosis. Previous study indicated that pre-treatment of simvastatin in rats before the ischemia-reperfusion induction significantly attenuated cardiac dysfunction and ameliorated coronary flow (Lefer et al. 1998). Other researches also indicated that simvastatin could increase autophagy in coronary arterial myocytes or cardiomyocytes by inhibition of major negative autophagy regulator Racl-mammalian target of rapamycin (mTOR), a pivotal AMPK/Akt downstream target (Wei et al. 2013; Andres et al. 2014). These findings indicated that simvastatin preserves the cardio protection effect for coronary heart diseases. The salient finding of our study revealed that high glucose contributes to cardiomyocytes dysfunction and injury with elevated apoptosis ratio, increased apoptosis related factor’s protein expressions and overt decreased autophagy. Results indicated that simvastatin could reverse the damages caused by high glucose administration, which attenuated apoptosis cell number and increased intracellular autophagy. The potential participation of autophagy in simvastatin treated high glucose toxicity was confirmed by pharmacological autophagy modulator 3-MA, which inhibited the autophagy induced by simvastatin. Taken together, our data revealed that simvastatin may attenuate high glucose induced injury in H9c2 cell by promoting intracellular autophagy. This finding further indicated that 3-MA negated simvastatin-offered amelioration by apoptosis and autophagy related protein expressions detection.

In this present study, H9c2 cells demonstrated increased apoptosis, which indicated that high glucose treatment leads to cell toxicity, and the results were in line with previous research (Rubin et al. 2012; Ouyang et al. 2014). Increased glucose level may contribute to cardiac apoptosis and the progression of diabetic cardiomyopathy (Huang Y-T et al. 2013). Caspase enzymes and Bcl-2 family are the main protein family that contribute to apoptosis (Thornberry and Lazebnik 1998). Bcl-2 protein is a cytosolic protein with a lipid anchoring domain, which allows it to target the nucleus and inhibit apoptosis. Under apoptosis-inducing stresses, Bcl-2 protein expression is markedly reduced in cardiac tissue (Xie et al. 2001). Bax, a member of Bcl-family, homodimerizes and forms heterodimers with Bcl-2 protein, which reduce the anti-apoptotic effect of Bcl-2 proteins, which is functionally opposed to Bcl-2 (Kroemer 1997). Using CCK-8 and western blotting assay, our data revealed that apoptosis overtly increased in response to high glucose exposure, the unfavourable effects of which was reversed by simvastatin. Despite apoptosis, autophagy is another critical process to maintain cellular homeostasis (Jafari Anarkooli et al. 2008). Autophagy plays an important role to keep cellular homeostasis in which long lived proteins and organelles are delivered to and cleared by lysosomes (Kobayashi and Liang 2015). Cardiac autophagy in animal is destructed by diet induced insulin resistance, metabolic syndrome and type II diabetes. In heart tissues, constitutive autophagy is a homeostatic mechanism for maintaining cardiac structure and function (Martinet et al. 2007). Previous studies has indicated that autophagy is destructed in type 1mice and type 2 diabetic animal heart tissues, which indicating that the autophagy deficiency may lead to diabetic cardiomyopathy (Xie et al. 2011; Guo et al. 2013; Zhao et al. 2013). Autophagy is characterized with several protein markers. Beclin-1 is a vital protein participated in nucleus complex formation and creates a section of double membrane (Huang et al. 2018). Autophagy-related gene (ATG) 5 is an early autophagosomal marker which transiently localized to punctate on mitochondria, followed by the late autophagosomal marker, light chain-3 (LC3) (Bento et al. 2016). During autophagy, LC3I, a cytosolic form of LC3, is conjugated to phosphatidylethanolamine to form LC3- phosphatidylethanolamine conjugate (LC3II), which is recruited to autophagosomal membranes, and the detection of this conversion (LC3I-LC3II) is commonly utilized to monitor autophagy because the amount of LC3II is directly correlated with the number of autophagosomes (Mizushima and Yoshimori 2007; Tanida et al. 2008). It is known that ATG5/ATG7 is essential for autophagy, which is related with microtubule associated LC3 truncation and lipidation, and may originate directly form the ER membrane and other membrane organelles (Kang et al. 2011). Inducible deletion of ATG5 in mice represents the loss of autophagy, which lead to heart failure (Nakai et al. 2007). The amount of p62 inversely correlated with autophagic flux, which can be quantified by immunoblotting assay or flow cytometry (Mizushima and Yoshimori 2007). In recent years, several studies suggested that statins have modulating effect for the regulation of autophagy and apoptosis (Yang et al. 2010; Ghavami et al. 2011). In our study, high glucose reduced autophagic flux compared with normal group cells was indicated by the variations of protein expressions of ATG5, Beclin1, p62 and LC3II/LC3I or the differences of immunofluorescence intensity of LC3. Our study revealed that simvastatin exhibited protective effect and was associated with restored autophagy. The protein expressions of ATG5 and Beclin and LC3II/LC3I were increased while p62 was reduced under the treatment of simvastatin, which indicated that autophagy ability of H9c2 cells were increased. Furthermore, 3-MA, an autophagy inhibitor, was utilized to evaluate autophagy flux, and reduced the protection effect of simvastatin which provided protection effect against cardiac toxicity under hyperglycaemia. It is also worth noticing that research indicated that oral administration of simvastatin mitigated injury caused by cardiopulmonary bypass, possibly by suppressing cardiac muscle autophagy (Hua et al. 2017). Based on various results, it is plausible for us to speculate that simvastatin functions differently in varying disease models and activates multiple signalling pathways to regulate autophagic action.

## Conclusions

For the first time, our study revealed that simvastatin attenuated high glucose induced injury by increasing autophagy in cardiomyocytes. However, further research is needed for the signal pathways underlining autophagy, such as the mTOR signal pathway and AMPK activity. Apoptosis and autophagy are regulated by a different group of regulators and molecules. However, this is also crosstalk between apoptosis and autophagy. Adenosine monophosphate-activated protein kinase (AMPK) and c-Jun N-terminal protein kinase 1 (JNK1) are reported to be the common signalling pathways by which apoptosis and autophagy are regulated (Jafari Anarkooli et al. 2008). Therefore, studies are also demanded for further research.
